# Diurnal Patterns of Gene Expression in the Dorsal Vagal Complex and the Central Nucleus of the Amygdala – Non-rhythm-generating Brain Regions

**DOI:** 10.3389/fnins.2020.00375

**Published:** 2020-05-11

**Authors:** Mary M. Staehle, Sean O’Sullivan, Rajanikanth Vadigepalli, Kate F. Kernan, Gregory E. Gonye, Babatunde A. Ogunnaike, James S. Schwaber

**Affiliations:** ^1^Department of Biomedical Engineering, Rowan University, Glassboro, NJ, United States; ^2^Department of Pathology, Anatomy, and Cell Biology, Daniel Baugh Institute for Functional Genomics and Computational Biology, Thomas Jefferson University, Philadelphia, PA, United States; ^3^Department of Chemical Engineering, University of Delaware, Newark, DE, United States

**Keywords:** diurnal rhythms, gene expression, dorsal vagal complex, central nucleus of the amygdala, qPCR

## Abstract

Genes that establish the circadian clock have differential expression with respect to solar time in central and peripheral tissues. Here, we find circadian-time-induced differential expression in a large number of genes not associated with circadian rhythms in two brain regions lacking overt circadian function: the dorsal vagal complex (DVC) and the central nucleus of the amygdala (CeA). These regions primarily engage in autonomic, homeostatic, and emotional regulation. However, we find striking diurnal shifts in gene expression in these regions of male Sprague Dawley rats with no obvious patterns that could be attributed to function or region. These findings have implications for the design of gene expression studies as well as for the potential effects of xenobiotics on these regions that regulate autonomic and emotional states.

## Introduction

Diurnal rhythm is a major factor in many aspects of mammalian function, including the regulation of gene expression (Reppert and Weaver, 2002; Sukumaran et al., 2010). In mammals, such diurnal regulation of expression is attributed to the presence of an endogenous circadian clock located in the suprachiasmatic nucleus (SCN) of the anterior hypothalamus that responds to solar time (Dibner et al., 2010). This circadian clock establishes and maintains the daily rhythms of physiological processes (Bugge et al., 2012). Perturbations in the circadian clock can lead to multiple disorders including cancer (Cadenas et al., 2014), major depressive disorder (Li et al., 2013), and alcoholic liver disease (Forsyth et al., 2015).

Peripheral tissues including the heart, kidney, liver and gastrointestinal tract also demonstrate gene expression variations based on circadian time (Oishi et al., 1998; Shieh, 2003; Lim et al., 2006; Hoogerwerf et al., 2007; Sládek et al., 2007; Hughes et al., 2009; Froy, 2011). For example, the pharmacokinetics and dynamics of xenobiotic metabolism in the liver have been shown to vary based on circadian time, thereby altering the balance between therapeutic response and toxicity (Paschos et al., 2010; Froy, 2011; Gachon and Firsov, 2011). Within the brain, most studies of clock-modulated gene expression effects have focused on the SCN. However, there also have been studies that have shown spatiotemporally controlled diurnal patterns of clock-controlled genes in the brain (e.g., Harbour et al., 2014), suggesting that although the SCN serves as master regulator of circadian rhythms, non-SCN brain regions also respond to and intone a diurnal rhythm. By most estimates, the number of diurnally-associated genes is modest; in mouse, it has been estimated that 5–25% of the transcriptome exhibits diurnal patterning (Panda et al., 2002; Li and Zhang, 2015). Here, we report widespread diurnal gene expression patterns in two non-rhythm-generating brain regions.

**FIGURE 1:**
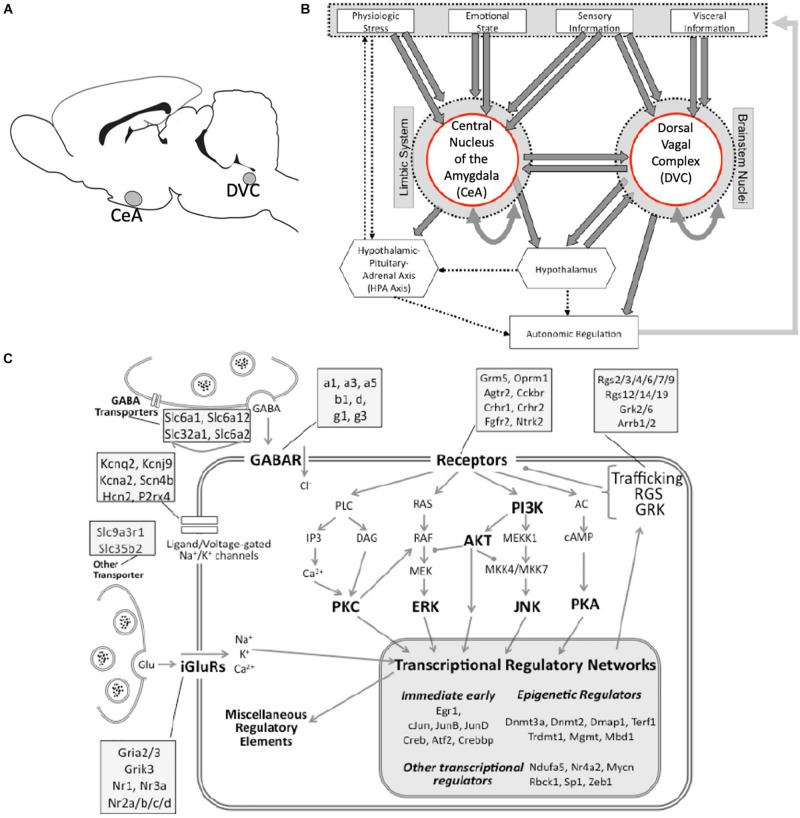
Neuroanatomical and neurobiological systems of interest. **(A)** Rat brain cartoon denoting sampled brain regions. CeA, central nucleus of the amygdala; DVC, Dorsal vagal complex. **(B)** A simplified cartoon representation showing the integrative roles of the CeA and the DVC in autonomic regulation. Direct connections are shown as heavy arrows, whereas indirect connections are shown as dotted arrows. Many anatomical and functional connections are omitted for clarity. **(C)** A graphical representation of the targeted cell biology including stress-responsive systems of excitatory and inhibitory neurotransmission, receptor-driven intracellular signaling, and transcription regulation (large shaded box). Major functional groups and signaling pathways are highlighted in bold. Compartments represented include the presynaptic terminal and membrane, and the post-synaptic membrane, cytoplasm and nucleus. Representative genes assigned to functional categories are listed in the attached shaded boxes.

The dorsal vagal complex (DVC) and the central nucleus of the amygdala (CeA) (Figure 1) are involved in receiving visceral inputs and generating autonomic outputs. We and others have found that the DVC, a major viscerosensory region in the brainstem, responds to a variety of inputs in order to maintain homeostasis in response to drugs, such as alcohol (Covarrubias et al., 2006; McDonald et al., 2008). Furthermore, the DVC has projections to the CeA that can modulate emotional state and behavior (Schwaber et al., 1982; Rosa et al., 2014). Moreover, the CeA is implicated in response to many drugs and plays an important role in addiction (Smith and Aston-Jones, 2008; Koob and Volkow, 2010; Roberto et al., 2012; de Guglielmo et al., 2019). We aimed to investigate the consequential influence these viscerosensory functions have on emotional state and behavior in the face of xenobiotic exposure. The focus herein stems from a multidimensional analysis of the resulting data, which revealed considerable patterns of diurnal variation in gene expression in the DVC and CeA. To the best of our knowledge, neither of these brain nuclei has been classified previously as rhythm-generating or solar-time-responsive like the SCN. However, it is reasonable to hypothesize that state change in these brain nuclei with respect to circadian time could have substantial influence on the subjective emotional experience of xenobiotics – particularly drugs of abuse and withdrawal from them.

Of the 145 transcripts measured in this study, 114 were determined to have a diurnal expression pattern in the DVC and/or the CeA. Of those, only 67 had diurnal patterns in both the DVC and the CeA, and only 22 of that subset were determined to have the same pattern in both brain regions. Taken together, this suggests broad spatiotemporally controlled effects of diurnal time on gene expression in the brain. Furthermore, diurnal gene expression patterns in the DVC and the CeA may underlie diurnal rhythms in physical and emotional regulation, which presents a potential mode of modulation by exogenous stressors that affect these systems. Ultimately, these results suggest that diurnal time should be controlled for explicitly in experimental designs involving gene expression in any brain tissue, and likely any *in vivo* tissue source.

## Materials and Methods

### Animals

All protocols were approved by the Institutional Animal Care and Use Committee of Thomas Jefferson University. Forty young, male, Sprague Dawley rats (35–45 g, Harlan Laboratories, Indianapolis, IN, United States) were housed individually in the Thomas Jefferson University Alcohol Research Center Animal Core Facility and fed standard chow and water until their weight reached 120 g (approximately 10 days). Upon achievement of the weight criteria, the rats were given an alcohol-free, maltose-dextrin substituted, Lieber DeCarli liquid diet (BioServ, Frenchtown, NJ, United States) *ad libitum* for 3 days. This diet is a liquid control diet that is nutritionally balanced to provide all necessary nutrients without supplementation (Lieber and Decarli, 1994). On subsequent days, each rat received a restricted quantity of the liquid diet such that the 24 h delayed caloric intake equaled the previous day’s intake of matched alcohol-fed animals. Only the calorie-restricted, alcohol-naïve animals are considered in this study. These animals and their alcohol-fed matches were also considered elsewhere (Freeman et al., 2012a, b, 2013). Rat weights were recorded weekly and linearly interpolated for intermediate days. The animal facility was maintained at constant temperature and humidity with a regular 12/12 light cycle [lights on at 07:00 (ZT0)].

The animals were sacrificed via rapid decapitation at one of three times of the day. For clarity, we use the standard circadian nomenclature of Zeitgeber time (ZT) to define the three times of the day, however, we note that this is not a study of circadian rhythms; the light cycles of all animals are identical and undisrupted. The three time points are: ZT3 (*n* = 7), ZT5 (*n* = 18), and ZT9 (*n* = 15). There were no differences among groups in the number of days on the calorie-restricted diet (ZT3: 39.71 ± 1.83 days; ZT5: 39.67 ± 0.95 days; ZT9: 39.07 ± 0.92 days; *p* > 0.3), and the overall rate of weight gain did not differ between groups (ZT3: 3.77 ± 0.39 g/day; ZT5: 3.73 ± 0.24 g/day; ZT9: 3.70 ± 0.24 g/day; *p* > 0.7). Thus, although these calorically-restricted animals may be different from standard chow-fed control animals, we consider all of the alcohol-naïve animals examined in this study to be experimentally equivalent to each other except for the Zeitgeber time of sacrifice; any baseline differences in expression due to caloric restriction are not examined herein.

### DVC and CeA Microdissection

Brains were excised immediately after decapitation and placed into ice-cold artificial cerebral spinal fluid (ACSF: 10 mM HEPES, pH 7.4; 140 mM NaCl; 5 mM KCl; 1 mM MgCl_2_; 1 mM CaCl_2_; 24 mM D-glucose). Brainstem and forebrain were separated and secured in individual agarose (4% UltraPure^TM^ low melting point agarose (Invitrogen, Carlsbad, CA, United States) in ACSF) blocks for slicing. Using a McIlwain Tissue Chopper (Gamshall, United Kingdom), the brainstem was sliced into 275 μm transverse sections, and the forebrain was sliced into 625 μm coronal sections. The slices were floated in ice-cold ACSF, and slices containing the regions of interest were selected. The DVC was identified and micropunched as previously reported (Khan et al., 2008). In an analogous manner, the CeA was identified by neuroanatomical landmarks defined by Paxinos and Watson (2007) and micropunched using size-matched micropunches (≤1.25 mm; Stoelting, Wood Dale, IL, United States). Bilateral punches were treated as a single sample. Each sample (two per animal: DVC and CeA) was tested separately and analyzed individually without pooling.

### High-Throughput qRT-PCR

RNA was extracted with either the RNeasy or the AllPrep DNA/RNA extraction kit (Qiagen, Valencia, CA, United States). Total RNA was DNAse treated (DNA-Free RNA kit, Zymo Research, Orange, CA, United States), RNA concentration and integrity were assessed with an ND-1000 (NanoDrop, Wilmington, DE, United States) and RNA nano 6000 chips on an Agilent 2100 Bioanalyzer, and the RNA was stored at −80°C. Reverse transcription was performed from 100 ng total RNA using SuperScript II (Invitrogen, Carlsbad, CA, United States).

A panel of genes representing various cellular processes and cellular localizations were developed for investigation (Supplementary Table S1). The panel is not exhaustive, and the genes were not selected with regard to circadian rhythm regulation nor caloric restriction, but rather were selected to provide a wide perspective of cellular activity (primarily for investigating the role of xenobiotic exposure). Cellular localization and function of the 145 genes are illustrated in Figure 1C. Intron-spanning PCR primers and probes were designed using Roche’s Universal Probe Library Assay Design Center^1^. The primers and probes for the 145 genes that passed quality control measures are listed in Supplementary Table S1. The standard BioMark^TM^ protocol was used to pre-amplify cDNA samples for 14 cycles (TaqMan^®^ PreAmp Master Mix: Applied Biosystems, Foster City, CA, United States). qRT-PCR reactions were performed with 96.96 BioMark^TM^ Dynamic Arrays (Fluidigm, South San Francisco, CA, United States), enabling simultaneous quantitative measurement of up to 96 transcripts in 96 samples each (Spurgeon et al., 2008; Orina et al., 2009). Runs were 40 cycles (15 s at 95°C; 5 s at 70°C; 60 s at 60°C). C_*T*_ values (Supplementary Tables S2, S3) were calculated using default settings in the Real-Time PCR Analysis Software (Fluidigm) and software-designated failed reactions were discarded from analysis.

### Experimental Design and Statistical Analysis

Fresh DVC and CeA samples were collected from rats immediately following rapid decapitation [ZT3 (*n* = 7), ZT5 (*n* = 18), and ZT9 (*n* = 15)]. RNA was extracted, reverse transcribed, and measured with microfluidic qPCR (see above). For each sample, the raw C_*T*_ values were quantile-normalized (ΔC_*T*_) in R version 2-11-1 (Bolstad et al., 2003). Quantile normalization has been shown to be viable alternative to normalization via an internal control, or housekeeping gene (Fujita et al., 2006; Pradervand et al., 2009). ΔΔC_*T*_ values were calculated for each gene by subtracting the mean ΔC_*T*_ at ZT5. −ΔΔC_*T*_ values were used for all visualizations of the relative expression changes across time points.

All statistical analyses were performed in R using the ΔC_*T*_ values. Time point and region effects were calculated with a standard 2-factor ANOVA model. Region-specific tests of time point effects were conducted with one-way ANOVA and both post-hoc two-tailed, Student’s *t*-tests and *post hoc* Tukey comparisons. Multidimensional scaling analysis (MDS) in three coordinate dimensions was conducted in R using *cmdscale*. All statistical testing was conducted at a 95% confidence level, and all stated errors are the 95% confidence intervals around the means. By nature of the quantity of genes and statistical comparisons (145 genes per statistical comparison type), we anticipate that these analyses will yield some false positives. However, we accept these errors without correction in order to describe the broad impacts of diurnal time. For each gene measured, the expression data, statistical analysis, and pattern determination (section Diurnal Pattern Classification) are provided in Supplementary Table S2 (DVC) and Supplementary Table S3 (CeA).

### Diurnal Pattern Classification

Genes were determined to have a diurnal expression pattern if: (1) the time point effect in the one-way ANOVA was significant (*p* ≤ 0.05); (2) *t*-test comparisons revealed a significant difference in gene expression between any two time points (ZT3 vs. ZT5, ZT5 vs. ZT9, or ZT3 vs. ZT9, *p* ≤ 0.05), *or* (3) the mean −ΔΔC_*T*_ value at any time point was greater than 0.25 or less than -0.25. These statistics are listed in Supplementary Tables S2, S3. Genes fulfilling at least one of these criteria were further classified into eight patterns according to the −ΔΔC_*T*_ magnitudes and the statistically significant differences between time points. Support for these patterns were recorded as either statistics-based (for those genes satisfying either or both of the first two criteria above) or threshold-based (for those genes satisfying only the third criterion). The threshold value of 0.25 was selected to capture the genes with larger average changes (∼20% or more) that are not selected by statistical criteria due to a high level of biological variability. There is no reason to expect that the DVC and the CeA have the same diurnal expression patterns, so each brain region’s pattern was determined separately.

### Alignment Score Calculation

In order to determine genes whose diurnal expression patterns differ between brain regions, we developed an alignment score based upon the calculated angle between two three-dimensional vectors representing the temporal expression patterns. For each gene, *m*, and each brain region, *i*, we calculated the mean ΔC_*T*_ value for each time point and combined the three values as a three-dimensional vector:

(1) $${\bar{x}}_{m,i}=\left(Z\,T\,3_{a\,v\,g\,\Delta \,C_{T},m,i},Z\,T\,5_{a\,v\,g\,\Delta \,C_{T},m,i},Z\,T\,9_{a\,v\,g\,\Delta \,C_{T},m,i}\right)$$

We then calculated an alignment score (AS) between the DVC and the CeA:

(2) $$AS_{m,D\,V\,C:C\,e\,A}^{*}=\frac{\left\langle {\bar{x}}_{m,DVC,}\,{\bar{x}}_{m,CeA,}\right\rangle }{{||{\bar{x}}_{m,D\,V\,C}||}^{*}\,||{\bar{x}}_{m,C\,e\,A}||}=\cos{\left(\theta \right)}_{m,D\,V\,C:C\,e\,A}$$

(3) $$A\,S_{m,D\,V\,C:C\,e\,A}=-\log_{10}\left(1-A\,S_{m,D\,V\,C:C\,e\,A}^{*}\right)$$

where the final transformation from *AS*^∗^ to *AS* in Equation 3 expands the scale to accentuate small changes in ΔC_*T*_ that are meaningful, but do not drastically alter the angle between the vectors, *θ*. With this transformation, a small (*AS*) indicates more divergent diurnal expression patterns, whereas a large *AS* indicates diurnal expression pattern similarity. Note that the denominator in Equation 2 ensures that any changes between brain regions that are not time-dependent do not influence the alignment score. Therefore, the alignment score is a quantitative measure of diurnal expression *pattern* changes in different brain regions.

## Results

### Multidimensional Scaling Shows Regional and Time Point Clustering

**FIGURE 2:**
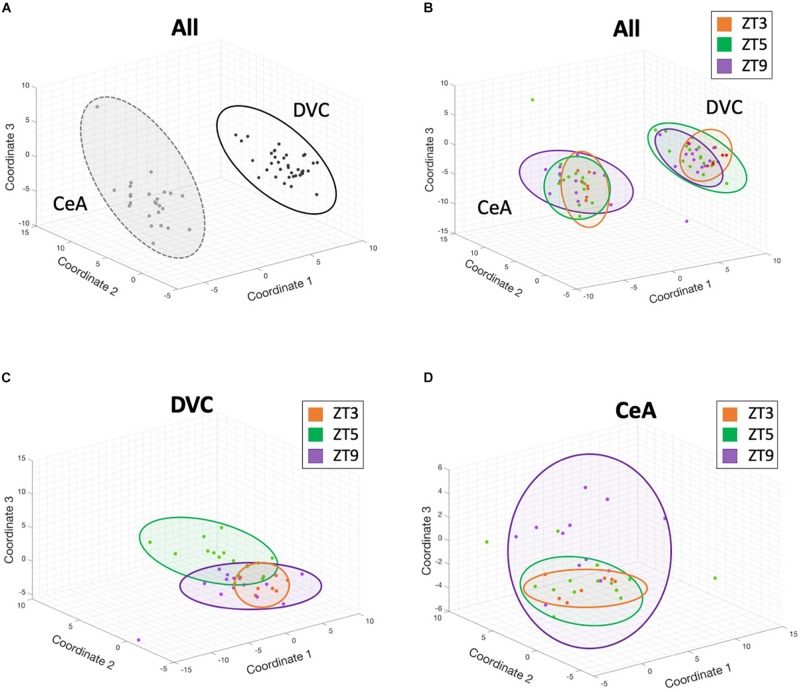
3-dimensional multidimensional scaling plots. Three-dimensional multidimensional scaling (MDS) analysis of gene expression from all genes and all samples reveals that brain region differences contribute the largest differentiating factor, but that diurnal time points within a single region cluster together in these complex dimensional coordinates. Each point represents the gene expression profile from a single sample. Distance between points in 3-dimensional space represents overall differences in gene expression between samples. **(A)** DVC samples (black) and CeA samples (gray) have different expression profiles. **(B)** Although brain region provides the largest differentiating factor when analyzing all samples, clustering by time point is also apparent. In panels B-D, ZT3 samples are shown in orange, ZT5 samples in green, and ZT9 samples in purple. **(C)** A separate multidimensional scaling analysis of only DVC samples shows complex separation of time points. **(D)** Similarly, a separate multidimensional scaling analysis of only CeA samples shows complex separation of time points. In all panels, colored ovals were added manually for visual guidance.

A multidimensional scaling (MDS) analysis (Figure 2) was performed to reduce the high-dimensional dataset into a visualization of the overall gene expression. Figure 2A shows that gene expression across the 145 genes assayed differed substantially between the DVC and CeA brain regions based on tight clustering of same-region samples. Samples from the same time point and region did show some clustering (Figure 2B), and the time point differences were accentuated when considering each region separately with subsequent region-only analyses (Figures 2C,D). These results suggest large and numerous gene expression differences between regions but also complex differences between time points within regions.

### Gene Expression Changes in the DVC and/or the CeA Across a 6 h Window

Two-factor ANOVA revealed significant time point effects for 43 of the 145 genes tested, indicating statistically significant changes in gene expression across the 6 h window from ZT3 to ZT9. However, the major source of expression variability in most genes was brain region; 107 of the 145 genes had a significant brain region effect with two-factor ANOVA (111 with one-way ANOVA), suggesting that brain region differences in gene expression may obscure other changes when the two regions are studied together. Subsequent intra-region one-way ANOVAs revealed 33 genes with significant time point effects in the DVC and 20 genes with significant time point effects in the CeA. Eight genes (Adrbk1, Agtrap, Creb1, Ptpn2, Mbd1, Nr4a2, Slc35b2, and Terf1) were found to have significant time point effects in both brain regions. *T*-tests revealed an additional 18 genes in the DVC and 8 genes in the CeA that had significant differences between time points (the full description for all genes can be found in Supplementary Table S4). In summary, 64 genes in the DVC and 54 genes in the CeA, for a total of 75 of the 145 genes tested had statistically significant gene expression changes in the DVC and/or the CeA due to diurnal variations.

### Diurnal Pattern Classification Reveals Asynchronous Dynamic Expression Patterns in the DVC and the CeA

**FIGURE 3:**
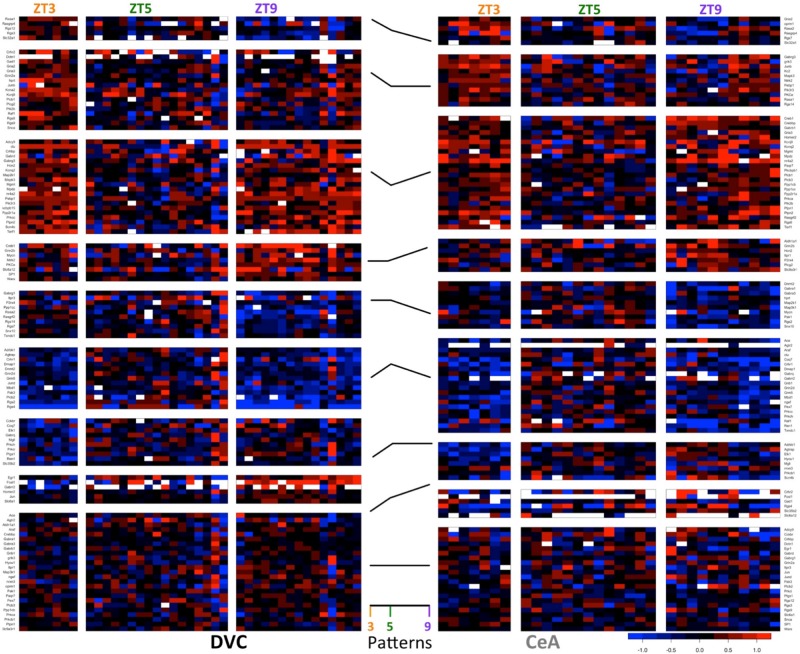
Diurnal patterns in gene expression in the DVC and CeA. Heatmaps of −ΔΔC_*T*_ values in DVC samples (left) and CeA samples (right) organized according to diurnal pattern (center). In the heatmaps, each *column* shows data from a single sample. For each brain region, samples are separated according to diurnal time (ZT3, ZT5, and ZT9). Each *row* shows the expression level of a single gene relative to the mean ZT5 expression in that brain region. Data for the 114 genes with a diurnal pattern in either the DVC or the CeA are shown. Two genes had patterns in the DVC, but expression levels were not detectable in the CeA. Therefore, only data from the remaining 112 genes are shown for the CeA.

In addition to the 64 genes found in the DVC with statistics-based patterns, 26 genes were determined to have only threshold-based diurnal expression patterns (3 genes demonstrated statistics-based patterns only). We normalized expression to the ZT5 time point for each gene resulting in eight possible non-zero gene expression patterns to characterize the gene expression dynamics across the three time points in this study (3^2^–1 = 8). There are at least 5 genes with each classified pattern in the DVC (Figure 3, *left*), indicating that diurnal expression patterns are asynchronous and complex.

Similarly, in the CeA (Figure 3, *right*), 38 genes demonstrated threshold-based expression patterns only (6 genes had statistics-based patterns only). The increase in threshold-based patterns in the CeA may indicate a greater degree of biological variability in CeA samples or a broader range of interactions between emotional state and basal gene expression rhythms.

### Functionality and Cellular Localization of Transcripts With Diurnal Patterns

**FIGURE 4:**
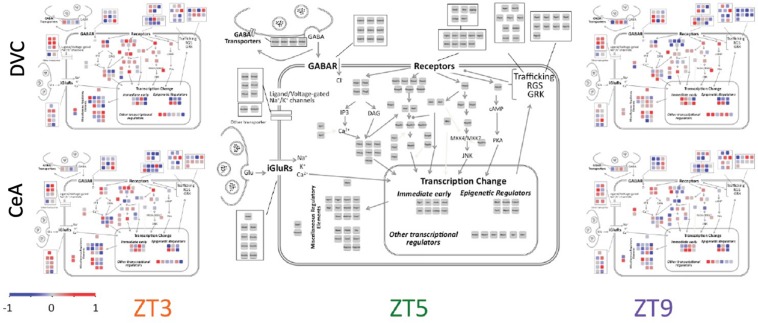
Cellular and signaling pathway view of diurnal gene expression changes. The center panel shows the genes measured in a layout matching that of Figure 1C in order to depict the gene expression according to the cellular and signaling pathways of interest. Average −ΔΔC_*T*_ values for each gene are used to colorize the subpanels using the color scale shown. The three columns show the three diurnal time points (ZT3, ZT5, and ZT9). The top row shows data from the DVC while the bottom row shows data from the CeA. By definition, the average −ΔΔC_*T*_ value of each gene at ZT5 is zero, and therefore the two brain regions share a common center panel.

Diurnal patterns of gene expression do not appear to be associated with cellular localization (Figure 4). Rather, transcripts in all parts of the cell showed large changes in expression across the 6 h period. Interestingly, some functionally related groups of transcripts have similar patterns, suggesting coordinated regulation of expression. For example, transcripts of regulators of G-protein signaling (Rgs proteins), such as those located in the top right corner in each panel of Figure 4, have similar diurnal regulation in the DVC, particularly at ZT9. Glutamate receptors (iGluR’s) have a variety of diurnal patterns that are relatively similar in the two brain regions, whereas the expression patterns of gamma-aminobutyric acid (GABA) receptors (GABAR) seem to be inverted between brain regions.

### Pattern Similarity and Differences Between the DVC and the CeA

**FIGURE 5:**
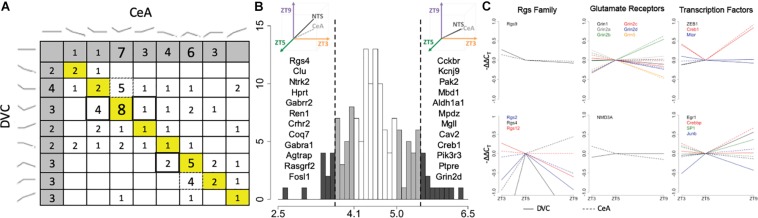
Pattern similarity and differences in the DVC and CeA. **(A)** Each cell shows the number of genes with the pattern combination found by matching the pattern in the DVC, *rows*, with the pattern in the CeA, *columns* (DVC patterns shown at left, CeA patterns shown at top). The 18 genes with the same pattern in the DVC and the CeA are included in the diagonal (yellow). Dotted box outlines highlight several cells with genes that have more diurnal dynamics in the CeA than in the DVC. Double box outlines highlight the reverse: cells with genes that have more diurnal dynamics in the DVC than in the CeA. The cells shaded in gray indicate transcripts with no diurnal pattern in the alternate brain region. **(B)** Histogram of the alignment scores of all 145 genes. Genes with low alignment scores (<3.7) like that illustrated at the top left and genes with high alignment scores (>5.5) like that illustrated in the top right are shaded in dark gray. The genes in these categories are listed in order of increasing alignment score within the corresponding region. Genes with moderately low (<4.1) and moderately high (>5.0) alignment scores are shaded in light gray. **(C)** The average −ΔΔC_*T*_ values of Rgs family members (column 1), glutamate receptors (column 2), and transcription factors (column 3) with low, moderately low, moderately high, or high alignment scores are shown. All genes within the group with high or moderately high alignment scores are shown in the top row, while all genes within the group with low or moderately low alignment scores are shown in the bottom row. Each gene is shown in a different color with solid lines representing the gene expression data from DVC samples and dotted lines representing the gene expression data from CeA samples.

Of the 145 transcripts measured, 114 were determined to have a diurnal expression pattern in the DVC and/or in the CeA. Of those, 67 had diurnal patterns in both the DVC and the CeA, and only 22 of that subset were determined to have the same pattern in both brain regions (Figure 5A). In order to quantify pattern similarity across the two brain regions, an alignment score was calculated for each gene based on the average expression at each time point (see section Materials and Methods). Genes with higher alignment scores have similar diurnal gene expression patterns in the DVC and the CeA, whereas genes with lower alignment scores have divergent patterns in the DVC and CeA. As shown in Figure 5B, 12 genes were identified to have a high alignment score (AS > 5.5, which represents approximately the top 10% of genes measured) and 12 different genes were identified to have a low alignment score (AS < 3.7, representing the lowest 10% of genes measured). An additional 23 genes with a moderately low alignment score (3.7 < AS < 4.1) and 22 genes with a moderately high alignment score (5.0 < AS < 5.5) were also identified. Three families of genes were observed to be of particular interest in this list of 67 genes: the regulators of G-protein signaling (Rgs) family, glutamate receptors, and transcription factors. As shown in Figure 5C, in general, Rgs family member transcripts had different diurnal expression patterns in the DVC and the CeA, indicating differential roles in these two brain regions in diurnal regulation, whereas glutamate receptors had very similar diurnal expression patterns in the two brain regions. For transcription factors, the relationships among gene expression patterns were complex: some had high alignment scores and some had low alignment scores.

## Discussion

Temporal gene expression patterns are well-studied in circadian research. Microarray experiments have led to the identification of genes with rhythmic circadian expression patterns in the SCN (Panda et al., 2002; Reppert and Weaver, 2002) and peripheral tissues (Panda et al., 2002; Storch et al., 2002). Other groups have studied the circadian rhythms of clock-related genes such as Per1, Per2, and Bmal1 in the CeA (Lamont et al., 2005; Amir and Robinson, 2006; Perrin et al., 2006; Segall et al., 2006; Ramanathan et al., 2008; Harbour et al., 2014) and in the DVC (Mieda et al., 2006; Kaneko et al., 2009) in rodents. However, we are not aware of any medium- or high-throughput studies of diurnal gene expression patterns in either the CeA or the DVC. The integral viscerosensory role of these nuclei implies that such diurnal gene expression patterns could be critical to first-person emotional experience in response to, for example, withdrawal from addictive substances. Thus, this phenomenon may play an important role in drug-seeking.

Discernable circadian rhythms in gene expression were found in less than 10% of all genes in the SCN, liver, and heart (Panda et al., 2002; Storch et al., 2002; Vollmers et al., 2009). Therefore, although we expected to find some genes with diurnal expression patterns, we were surprised to find that more than half of the genes tested in each brain region had at least one statistically significant difference between two individual time points. We note that these high percentages depend on the subset of genes assayed, and may not be representative of global expression in the CeA and DVC. We speculate this could result from overrepresentation of inter- and intra-cellular signaling-involved genes. Furthermore, the patterns of gene expression we describe do not include phasic patterns that are characteristic of circadian expression patterns – three time points across a 9 h period provides a resolution too low to resolve phasic patterning. These experiments were not designed to capture circadian expression patterns and we cannot deduce such patterning from these data. However, the groups of animals at different time points were not different in any respect, except the time of day at which they were sacrificed. Genes assayed were not chosen as circadian rhythm regulators or as those known to play a role in generating diurnal rhythms, yet the expression of these genes showed distinct time-of-day patterns at the three time points investigated. Because a high percentage of the genes assayed demonstrated this disparate expression behavior in brain regions critical to physical and emotional homeostatic regulation, we conjecture that similar expression patterns likely exist throughout the brain, and this kind of “hidden variability” could have considerable impact on the design and execution of gene expression experiments. Additionally, it is prudent to keep in mind that the scope of this study was limited to all male animals on a calorie-restricted diet. Differential expression with respect to diurnal rhythms may be profoundly influenced by hormonal signaling or other physiologic processes that differ between the sexes. Further, calorie restriction is known to influence the circadian clock itself (Froy, 2007). While these limitations may reduce generalizability of the results, they should not lessen the impact of the general suggestions of these findings.

The results from our present study suggest that the DVC and CeA, which are critical in autonomic and emotional regulation, implement time-dependent gene expression patterns that underlie their baseline regulatory activity. We conjecture that these pattern changes would affect corresponding metabolic or protein enrichment changes, but in this study, we are unable to assess downstream effects. Nonetheless, these results serve as cautionary advice for subsequent gene expression studies in the brain: diurnal time should be considered explicitly in experimental design regardless of brain region for meaningful comparisons. Furthermore, these data lay a foundation for investigating xenobiotic-induced homeostatic or emotional disturbances due to diurnal changes in gene expression patterns in the CeA and DVC beyond any global changes induced by these perturbations. Diurnal gene expression variations in these nuclei may influence the physical dependence or addictive properties of certain drugs. Conversely, xenobiotics may influence the diurnal rhythms in these nuclei such that dysregulation occurs leading to negative emotional states or behavior change.

## Data Availability Statement

The datasets generated in this study are publicly available on the Gene Expression Omnibus (https://www.ncbi.nlm.nih.gov/geo/query/acc.cgi?acc=GSE147569), under the accession no. GSE147569.

## Ethics Statement

The animal study was reviewed and approved by the Institutional Animal Care and Use Committee of Thomas Jefferson University.

## Author Contributions

MS designed the study and conducted experiments, performed analysis, generated figures, and was a main contributor to the writing of the manuscript. SO’S performed analysis, generated figures, and was a main contributor to the writing of the manuscript. RV, GG, and JS designed the study and were involved in analysis, figure design, and editing. KK and BO were involved in analysis and editing. All authors discussed the results and commented on the manuscript.

## Conflict of Interest

The authors declare that the research was conducted in the absence of any commercial or financial relationships that could be construed as a potential conflict of interest.

## Footnotes

1 www.universalprobelibrary.com

## References

[B1] Amir S. Robinson B. (2006). Thyroidectomy alters the daily pattern of expression of the clock protein, PER2, in the oval nucleus of the bed nucleus of the stria terminalis and central nucleus of the amygdala in rats. *Neurosci. Lett.* 407 254–257. 10.1016/j.neulet.2006.08.057 16973268

[B2] Bolstad B. M. Irizarry R. A. Åstrand M. Speed T. P. (2003). A comparison of normalization methods for high density oligonucleotide array data based on variance and bias. *Bioinformatics* 19, 185–193. 10.1093/bioinformatics/19.2.185 12538238

[B3] Bugge A. Feng D. Everett L. J. Briggs E. R. Mullican S. E. Wang F. (2012). Rev-erbα and Rev-erbβ coordinately protect the circadian clock and normal metabolic function. *Genes Dev.* 26 657–667. 10.1101/gad.186858.112 22474260 PMC3323877

[B4] Cadenas C. van de Sandt L. Edlund K. Lohr M. Hellwig B. Marchan R. (2014). Loss of circadian clock gene expression is associated with tumor progression in breast cancer. *Cell Cycle* 13 3282–3291. 10.4161/15384101.2014.954454 25485508 PMC4613905

[B5] Covarrubias M. Y. Khan R. L. Vadigepalli R. Hoek J. B. Schwaber J. S. (2006). Chronic alcohol exposure alters transcription broadly in a key integrative brain nucleus for homeostasis: the nucleus tractus solitarius. *Physiol. Genomics* 24 45–58. 10.1152/physiolgenomics.00184.2005 16189278

[B6] de Guglielmo G. Kallupi M. Pomrenze M. B. Crawford E. Simpson S. Schweitzer P. (2019). Inactivation of a CRF-dependent amygdalofugal pathway reverses addiction-like behaviors in alcohol-dependent rats. *Nat. Commun.* 10 1–11. 10.1038/s41467-019-09183-0 30886240 PMC6423296

[B7] Dibner C. Schibler U. Albrecht U. (2010). The mammalian circadian timing system: organization and coordination of central and peripheral clocks. *Annu. Rev. Physiol.* 72 517–549. 10.1146/annurev-physiol-021909-135821 20148687

[B8] Forsyth C. B. Voigt R. M. Burgess H. J. Swanson G. R. Keshavarzian A. (2015). Circadian rhythms, alcohol and gut interactions. *Alcohol* 49 389–398. 10.1016/j.alcohol.2014.07.021 25499101 PMC4431951

[B9] Fujita A. Sato J. R. de Oliveira Rodrigues L. Ferreira C. E. Sogayar M. C. (2006). Evaluating different methods of microarray data normalization. *BMC Bioinformatics* 7:469 10.1186/1471-2105-7-469 PMC1636075 17059609

[B10] Freeman K. Brureau A. Vadigepalli R. Staehle M. M. Brureau M. M. Gonye G. E. (2012a). Temporal changes in innate immune signals in a rat model of alcohol withdrawal in emotional and cardiorespiratory homeostatic nuclei. *J. Neuroinflamm.* 9:97. 10.1186/1742-2094-9-97 22626265 PMC3411448

[B11] Freeman K. Staehle M. M. Gümüş Z. H. Vadigepalli R. Gonye G. E. Nichols C. N. (2012b). Rapid temporal changes in the expression of a set of neuromodulatory genes during alcohol withdrawal in the dorsal vagal complex: molecular evidence of homeostatic disturbance. *Alcohol. Clin. Exp. Res.* 36 1688–1700. 10.1111/j.1530-0277.2012.01791.x 22486438 PMC4419739

[B12] Freeman K. Staehle M. M. Vadigepalli R. Gonye G. E. Ogunnaike B. A. Hoek J. B. (2013). Coordinated dynamic gene expression changes in the central nucleus of the amygdala during alcohol withdrawal. *Alcohol. Clin. Exp. Res.* 37 E88–E100. 10.1111/j.1530-0277.2012.01910.x 22827539 PMC4408903

[B13] Froy O. (2007). The relationship between nutrition and circadian rhythms in mammals. *Front. Neuroendocrinol.* 28 61–71. 10.1016/j.yfrne.2007.03.001 17451793

[B14] Froy O. (2011). The circadian clock and metabolism. *Clin. Sci.* 120 65–72. 10.1042/cs20100327 20929440

[B15] Gachon F. Firsov D. (2011). The role of circadian timing system on drug metabolism and detoxification. *Expert Opin. Drug Metab. Toxicol.* 7 147–158. 10.1517/17425255.2011.544251 21192771

[B16] Harbour V. L. Weigl Y. Robinson B. Amir S. (2014). Phase differences in expression of circadian clock genes in the central nucleus of the amygdala, dentate gyrus, and suprachiasmatic nucleus in the rat. *PLoS One* 9:e103309 10.1371/journal.pone.0103309 PMC4113347 25068868

[B17] Hoogerwerf W. A. Hellmich H. L. Cornélissen G. Halberg F. Shahinian V. B. Bostwick J. (2007). Clock gene expression in the murine gastrointestinal tract: endogenous rhythmicity and effects of a feeding regimen. *Gastroenterology* 133 1250–1260. 10.1053/j.gastro.2007.07.009 17919497

[B18] Hughes M. E. DiTacchio L. Hayes K. R. Vollmers C. Pulivarthy S. Baggs J. E. (2009). Harmonics of circadian gene transcription in mammals. *PLoS Genet.* 5:e1000442 10.1371/journal.pgen.1000442 PMC2654964 19343201

[B19] Kaneko K. Yamada T. Tsukita S. Takahashi K. Ishigaki Y. Oka Y. (2009). Obesity alters circadian expressions of molecular clock genes in the brainstem. *Brain Res.* 1263 58–68. 10.1016/j.brainres.2008.12.071 19401184

[B20] Khan R. L. Vadigepalli R. McDonald M. K. Rogers R. F. Gao G. R. Schwaber J. S. (2008). Dynamic transcriptomic response to acute hypertension in the nucleus tractus solitarius. *Am. J. Physiol. Regul. Integr. Compar. Physiol.* 295 R15–R27. 10.1152/ajpregu.00152.2008 18434436 PMC2494808

[B21] Koob G. F. Volkow N. D. (2010). Neurocircuitry of addiction. *Neuropsychopharmacology* 35 217–238. 10.1038/npp.2009.110 19710631 PMC2805560

[B22] Lamont E. W. Robinson B. Stewart J. Amir S. (2005). The central and basolateral nuclei of the amygdala exhibit opposite diurnal rhythms of expression of the clock protein Period2. *Proc. Natl. Acad. Sci. U.S.A.* 102 4180–4184. 10.1073/pnas.0500901102 15746242 PMC554834

[B23] Li J. Z. Bunney B. G. Meng F. Hagenauer M. H. Walsh D. M. Vawter M. P. (2013). Circadian patterns of gene expression in the human brain and disruption in major depressive disorder. *Proc. Natl. Acad. Sci. U.S.A.* 110 9950–9955. 10.1073/pnas.1305814110 23671070 PMC3683716

[B24] Li S. Zhang L. (2015). Circadian control of global transcription. *BioMed Res. Int.* 2015:187809. 10.1155/2015/187809 PMC4670846 26682214

[B25] Lieber C. S. Decarli L. M. (1994). Animal models of chronic ethanol toxicity. *Methods Enzymol.* 233 585–594. 10.1016/s0076-6879(94)33061-1 8015491

[B26] Lim F. L. Currie R. A. Orphanides G. Moggs J. G. (2006). Emerging evidence for the interrelationship of xenobiotic exposure and circadian rhythms: a review. *Xenobiotica* 36 1140–1151. 10.1080/00498250600861819 17118921

[B27] McDonald M. Hoek J. Ogunnaike B. Schwaber J. (2008). Behavioral and neurobiological changes within a period of heightened susceptibility to voluntary alcohol withdrawal. *FASEB J. Federat. Am. Soc. Exp. Biol.* 22 (1 Suppl.), 946.7–946.7.

[B28] Mieda M. Williams S. C. Richardson J. A. Tanaka K. Yanagisawa M. (2006). The dorsomedial hypothalamic nucleus as a putative food-entrainable circadian pacemaker. *Proc. Natl. Acad. Sci. U.S.A.* 103 12150–12155. 10.1073/pnas.0604189103 16880388 PMC1567710

[B29] Oishi K. Sakamoto K. Okada T. Nagase T. Ishida N. (1998). Antiphase circadian expression between BMAL1 and period homologue mRNA in the suprachiasmatic nucleus and peripheral tissues of rats. *Biochem. Biophys. Res. Commun.* 253 199–203. 10.1006/bbrc.1998.9779 9878515

[B30] Orina J. N. Calcagno A. M. Wu C. P. Varma S. Shih J. Lin M. (2009). Evaluation of current methods used to analyze the expression profiles of ATP-binding cassette transporters yields an improved drug-discovery database. *Mol. Cancer Ther.* 8, 2057–2066. 10.1158/1535-7163.MCT-09-0256 19584229 PMC2736804

[B31] Panda S. Antoch M. P. Miller B. H. Su A. I. Schook A. B. Straume M. (2002). Coordinated transcription of key pathways in the mouse by the circadian clock. *Cell* 109 307–320. 10.1016/s0092-8674(02)00722-5 12015981

[B32] Paschos G. K. Baggs J. E. Hogenesch J. B. FitzGerald G. A. (2010). The role of clock genes in pharmacology. *Annu. Rev. Pharmacol. Toxicol.* 50 187–214. 10.1146/annurev.pharmtox.010909.105621 20055702

[B33] Paxinos G. Watson C. (2007). *The rat brain in stereotaxic coordinates*, 6 Edn. Amsterdam: Academic. 10.1016/0165-0270(80)90021-7 6110810

[B34] Perrin J. S. Segall L. A. Harbour V. L. Woodside B. Amir S. (2006). The expression of the clock protein PER2 in the limbic forebrain is modulated by the estrous cycle. *Proc. Natl. Acad. Sci. U.S.A.* 103 5591–5596. 10.1073/pnas.0601310103 16554373 PMC1459398

[B35] Pradervand S. Weber J. Thomas J. Bueno M. Wirapati P. Lefort K. (2009). Impact of normalization on miRNA microarray expression profiling. *RNA* 15, 493–501. 10.1261/rna.1295509 19176604 PMC2657010

[B36] Ramanathan C. Nunez A. A. Smale L. (2008). Daily rhythms in PER1 within and beyond the suprachiasmatic nucleus of female grass rats (*Arvicanthis niloticus*). *Neuroscience* 156 48–58. 10.1016/j.neuroscience.2008.07.020 18692118 PMC2758417

[B37] Reppert S. M. Weaver D. R. (2002). Coordination of circadian timing in mammals. *Nature* 418 935–941. 10.1038/nature00965 12198538

[B38] Roberto M. Gilpin N. W. Siggins G. R. (2012). The central amygdala and alcohol: role of γ-aminobutyric acid, glutamate, and neuropeptides. *Cold Spring Harb. Perspect. Med.* 2:a012195. 10.1101/cshperspect.a012195 23085848 PMC3543070

[B39] Rosa J. Myskiw J. C. Furini C. R. Sapiras G. G. Izquierdo I. (2014). Fear extinction can be made state-dependent on peripheral epinephrine: role of norepinephrine in the nucleus tractus solitarius. *Neurobiol. Learn. Mem.* 113 55–61. 10.1016/j.nlm.2013.09.018 24161888

[B40] Schwaber J. S. Kapp B. S. Higgins G. A. Rapp P. R. (1982). Amygdaloid and basal forebrain direct connections with the nucleus of the solitary tract and the dorsal motor nucleus. *J. Neurosci.* 2 1424–1438. 10.1523/jneurosci.02-10-01424.1982 6181231 PMC6564412

[B41] Segall L. A. Perrin J. S. Walker C. D. Stewart J. Amir S. (2006). Glucocorticoid rhythms control the rhythm of expression of the clock protein, Period2, in oval nucleus of the bed nucleus of the stria terminalis and central nucleus of the amygdala in rats. *Neuroscience* 140 753–757. 10.1016/j.neuroscience.2006.03.037 16678973

[B42] Shieh K. R. (2003). Distribution of the rhythm-related genes rPERIOD1, rPERIOD2, and rCLOCK, in the rat brain. *Neuroscience* 118 831–843. 10.1016/s0306-4522(03)00004-6 12710990

[B43] Sládek M. Rybová M. Jindráková Z. Zemanová Z. Polidarová L. Mrnka L. (2007). Insight into the circadian clock within rat colonic epithelial cells. *Gastroenterology* 133 1240–1249. 10.1053/j.gastro.2007.05.053 17675004

[B44] Smith R. J. Aston-Jones G. (2008). Noradrenergic transmission in the extended amygdala: role in increased drug-seeking and relapse during protracted drug abstinence. *Brain Struct. Funct.* 213 43–61. 10.1007/s00429-008-0191-3 18651175 PMC3632504

[B45] Spurgeon S. L. Jones R. C. Ramakrishnan R. (2008). High throughput gene1 expression measurement with real time PCR in a microfluidic dynamic array. *PLoS One* 3:e1662 10.1371/journal.pone.0001662 PMC2244704 18301740

[B46] Storch K. F. Lipan O. Leykin I. Viswanathan N. Davis F. C. Wong W. H. (2002). Extensive and divergent circadian gene expression in liver and heart. *Nature* 417 78–83. 10.1038/nature744 11967526

[B47] Sukumaran S. Almon R. R. DuBois D. C. Jusko W. J. (2010). Circadian rhythms in gene expression: relationship to physiology, disease, drug disposition and drug action. *Adv. Drug Deliv. Rev.* 62 904–917. 10.1016/j.addr.2010.05.009 20542067 PMC2922481

[B48] Vollmers C. Gill S. DiTacchio L. Pulivarthy S. R. Le H. D. Panda S. (2009). Time of feeding and the intrinsic circadian clock drive rhythms in hepatic gene expression. *Proc. Natl. Acad. Sci. U.S.A.* 106 21453–21458. 10.1073/pnas.0909591106 19940241 PMC2795502

